# High Prevalence of Human Polyomavirus 7 in Cholangiocarcinomas and Adjacent Peritumoral Hepatocytes: Preliminary Findings

**DOI:** 10.3390/microorganisms8081125

**Published:** 2020-07-27

**Authors:** Faisal Klufah, Ghalib Mobaraki, Emil Chteinberg, Raed A. Alharbi, Véronique Winnepenninckx, Ernst Jan M. Speel, Dorit Rennspiess, Steven W. Olde Damink, Ulf P. Neumann, Anna Kordelia Kurz, Iryna Samarska, Axel zur Hausen

**Affiliations:** 1 Department of Pathology, GROW-School for Oncology & Developmental Biology, Maastricht University, Medical Centre+, 6229 HX Maastricht, The Netherlands; faisal.klufah@mumc.nl (F.K.); g.mobaraki@maastrichtuniversity.nl (G.M.); emil.chteinberg@mumc.nl (E.C.); v.winnepenninckx@mumc.nl (V.W.); ernstjan.speel@mumc.nl (E.J.M.S.); dorit.rennspiess@mumc.nl (D.R.); iryna.samarska@mumc.nl (I.S.); 2 Department of Laboratory Medicine, Faculty of Applied Medical Sciences, Albaha University, Albaha 65779, Saudi Arabia; ralharbi@bu.edu.sa; 3 Department of Medical Laboratories Technology, Faculty of Applied Medical Sciences, Jazan University, Jazan 45142, Saudi Arabia; 4 Department of Surgery, NUTRIM School of Nutrition and Translational Research in Metabolism, Maastricht University, 6200 MD Maastricht, The Netherlands; steven.oldedamink@maastrichtuniversity.nl (S.W.O.D.); uneumann@ukaachen.de (U.P.N.); 5 Department of General Visceral and Transplantation Surgery, RWTH University Hospital Aachen, 52074 Aachen, Germany; 6 Department of Internal Medicine IV, RWTH Aachen University Hospital, 52074 Aachen, Germany; kordelia.zurhausen@gmx.de

**Keywords:** HPyV7, HPyV6, small DNA viruses, non-neoplastic hepatocytes, cancer, bile duct, Merkel cell polyomavirus, tumorigenesis

## Abstract

Cholangiocarcinoma (CCA) is a rare biliary-duct malignancy with poor prognosis. Recently, the presence of the human polyomavirus 6 (HPyV6) has been reported in the bile of diverse hepatobiliary diseases, particularly in the bile of CCA patients. Here, we investigated the presence of novel HPyVs in CCA tissues using diverse molecular techniques to assess a possible role of HPyVs in CCA. Formalin-Fixed Paraffin-Embedded (FFPE) tissues of 42 CCA patients were included in this study. PCR-based screening for HPyVs was conducted using degenerated and HPyV-specific primers. Following that, we performed FISH, RNA in situ hybridization (RNA-ISH), and immunohistochemistry (IHC) to assess the presence of HPyVs in selected tissues. Of all 42 CCAs, 25 (59%) were positive for one HPyV, while 10 (24%) CCAs were positive for 2 HPyVs simultaneously, and 7 (17%) were negative for HPyVs. Of the total 35 positive CCAs, 19 (45%) were positive for HPyV7, 4 (9%) for HPyV6, 2 (5%) for Merkel cell polyomavirus (MCPyV), 8 (19%) for both HPyV7/MCPyV, and 2 (5%) for both HPyV6/HPyV7 as confirmed by sequencing. The presence of viral nucleic acids was confirmed by specific FISH, while the RNA-ISH confirmed the presence of HPyV6 on the single-cell level. In addition, expression of HPyV7, HPyV6, and MCPyV proteins were confirmed by IHC. Our results strongly indicate that HPyV7, HPyV6, and MCPyV infect bile duct epithelium, hepatocytes, and CCA cells, which possibly suggest an indirect role of these viruses in the etiopathogenesis of CCA. Furthermore, the observed hepatotropism of these novel HPyV, in particular HPyV7, might implicate a role of these viruses in other hepatobiliary diseases.

## 1. Introduction

Cholangiocarcinoma (CCA) is a rare but very aggressive biliary duct neoplasia with a very poor prognosis [1]. The etiology of CCA is still largely obscure. However, several risk factors have been identified to be associated with the etiopathogenesis of CCA, e.g., primary sclerosing cholangitis, hepatolithiasis, inflammatory bowel disease, cirrhosis, diabetes, and biliary-duct cysts. Additionally, the role of environmental factors, e.g., toxins and smoking and microbiological infections, including hepatic parasites (flukes), and Hepatitis B and C viruses has been established in the etiopathogenesis of CCA [1,2,3]. Although CCA is rather uncommon in Western countries, the incidence of CCA has been steadily–yet inexplicably—rising during the last few decades [1]. Chan et al. recently reported the presence of the novel human polyomavirus 6 (HPyV6) in the bile of Chinese patients with diverse hepatobiliary diseases [4]. Human polyomaviruses (HPyVs) are small DNA viruses in which e.g., BK polyomavirus (BKPyV) and JC polyomavirus (JCPyV) have been identified since the early 1970s [5,6]. The current number of members of HPyVs has recently been expanded to fourteen, most of which have been identified within the past twelve years [7,8]. However, the only HPyV that has been identified as a human tumor virus is the Merkel cell polyomavirus (MCPyV) [9]. Approximately 80% of Merkel cell carcinoma (MCC) cases are caused by clonal integration of MCPyV in the tumor genome of this highly malignant MCC. In addition, MCPyV-positive MCCs harbor tumor-specific oncogenic mutations in the viral oncogene encoding large tumor antigen (LTAg) [7,10,11,12,13]. Here, we assessed the prevalence of human polyomavirus 7 (HPyV7), HPyV6, and the oncogenic MCPyV in paraffin-embedded tissue sections (FFPE) of patients diagnosed with CCA, using diverse molecular techniques.

## 2. Materials and Methods

### 2.1. Patients and Specimens

Forty-two formalin fixed and paraffin embedded (FFPE) tissue specimens of CCA patients collected at the Department of Pathology, Maastricht University Medical Centre+ (Maastricht Pathology Tissue Collection number: 2015-13) were included in this study. The study was approved by the Medical Ethics Review Committee of the Maastricht UMC+, the Netherlands (2019-0977), all tissue resections were collected and studied in accordance with the protocol of the Dutch Code of Conduct for Observational Research with Personal Data (2004) and Tissue [14].

Of these 42 CCA patients, 25 (60%) were male and 17 (40%) female. The mean age was 65.2 years (range: 29–85 yrs.). Three experienced pathologists (I.S., V.W., and A.z.H.) reviewed the histopathological and cytopathological features, and a consensus diagnosis of CCA subtypes was established. The recognition of the tubular non-neoplastic bile duct epithelium and neoplastic CCA cells has been performed by the above-mentioned pathologists.

DNA was isolated from all 42 FFPE tissues using techniques as previously described [15]. In brief, tissue sections were deparaffinized with xylene and the DNA was then extracted using the protocol of Genomic DNA from a tissue kit by Macherey-Nagel. The DNA concentration was assessed using a spectrophotometer (NanoDrop 2000, Thermo Scientific™, Wilmington, DE, USA). Per sample, 250 ng was added in a PCR reaction. All isolated DNAs were assessed for quality and integrity using multiplex primers (SCS: specimen control size) as described previously [15,16].

### 2.2. Degenerated PCR

In order to screen for the presence of HPyVs DNA, we modified the degenerated primers as recently published by Chan et al. [4]. The primer modification was performed according to the multiple alignment of the conservative region which is located within the conserved region in LTAg of HPyVs genome (Supplementary Figure S4a,b). To validate these degenerate primer sets, diverse PyVs-carrying plasmids were used, in addition to the MCPyV-positive MCC cell line (MKL-1) as positive controls in the PCR analysis. Bio-performance certified water was used as a non-template negative control.

The first nineteen isolated DNAs were screened for the presence of HPyVs DNA using degenerate primers, followed by sequencing of the obtained PCR products. DNA sequences were compared with the reference sequences of the National Center for Biotechnology Information (NCBI) Entrez Nucleotide database using the NCBI Blast program.

### 2.3. Specific HPyVs DNA PCR

In addition to the degenerated PCR technique, the specific PCRs targeting different gene regions of HPyV7, HPyV6, and MCPyV were performed using the protocols as previously described [15]. We used PCR to test all 42 CCA DNAs to amplify small tumor antigen (sTAg) and large tumor antigen (LTAg) of HPyV7, while sTAg genes of HPyV6 were amplified, as well as for three MCPyV genes, i.e., LT3, VP1, and M1/M2. In addition, multiple sequence alignment was performed for each primer result using the Clustal Omega algorithm (by The European Bioinformatics Institute) to compare with the positive control sequencing in the reference sequences of NCBI. All HPyV specific primers used in this study are summarized in Table S2.

### 2.4. Fluorescence In Situ Hybridization (FISH)

All FISH procedures were carried out as described previously [15,17,18,19]. Additionally, to check whether or not the FISH protocol earlier described by Hopman et al. was optimal for our CCA FFPE tissues cohort, we hybridized selected tissues with the alpha satellite (centromeric) DNA probe (Vysis-CEP 12 by Abbott Co., Abbott Park, IL, USA) to ensure the quality of the pretreatment for both parenchymal and non-parenchymal liver cells. The majority of FFPE specimens that were tested positive by DNA PCR for one of the HPyVs were tested by DNA FISH for the presence of HPyV6, or 7 or MCPyV to confirm the presence of the DNA in single cell level with an alternative technique. In addition, we evaluated and validated the viral DNA signals to confirm if the signals were specific. We treated the positive controls and selected CCA tissues from our cohort with a FISH protocol in combination with either DNase I or RNAse prior to hybridization. By adding DNase then retesting, we ensured that the signal captured was DNA-specific; and by adding RNase, we ensured that the signal was not RNA-specific. Positive controls revealed specific signals that diminished slightly when the tissue was treated with RNAse prior to hybridization. However, all specific viral DNA signals vanished completely in the positive controls when treated with DNAse I (Supplementary Figure S3a) prior to hybridization. No cross-reactivity for the probes of HPyV 6 or 7 or MCPyV was observed. In addition, tissues previously tested negative for viral DNA served as negative controls for FISH, and no signals were seen. Furthermore, no specific nuclear signals were generated by omission of the probe during the hybridization. Therefore, HPyV6, HPyV7, and MCPyV biotin probes appeared to be sufficiently specific to detect the specific virus DNA (Supplementary Figure S3a).

The FISH slides were analyzed by at least three members (F.K., E.J.S., and A.z.H.) and the results of all cases were discussed together with all authors according to the criteria as previously described by Hafkamp et al. [20]. In addition, the specificity of the FISH probes was assessed on the liver tissues, by both DNase I and RNAse treatment, as described above (Supplementary Figure S3b).

### 2.5. RNA In Situ Hybridization (RISH)

The RNAscope assay using complementary RNA probes for the localization of specific expression of HPyVs mRNA was applied to selected cases: A specific probe for both HPyV6 and 7 LTAg genes was designed by Advanced Cell Diagnostics (ACD). A total of 20 pairs of 50 bp pooled probe designed for the HPyV7 target region between 3688 and 4876 bp (V-Polyomavirus-HPyV7, Accession No: HM011566.1). Another 20 pairs of RNA probes for the HPyV6 target region 3786–4898 bp (V-Polyomavirus-HPyV6, Accession No: HM011563.1). The sections were pretreated using the RNAscope^®^ HD Red 2·5 Kit (Advanced Cell Diagnostics, Cat No. 322350, Newark, CA, USA) according to the manufacturer’s instructions.

A probe for human peptidylprolyl isomerase B (Hs-PPIB) mRNA expression was used as a positive control, while a probe for bacterial dihydrodipicolinate reductase gene (DapB) expression was used as a negative control. First, Hs-PPIB and DapB probes were applied to FFPE HeLa cells to validate the pretreatment and hybridization conditions according to ACD protocols [21]. In addition, the two control probes were applied to each tested CCA case to confirm the expression of Hs-PPIB RNA and no expression of DapB. All RISH slides were evaluated and graded according to the ACD protocols and photos were taken using the VENTANA iScan-HT slide scanner [21].

### 2.6. Immunohistochemistry (IHC)

The following antibodies and dilutions were used: the monoclonal antibodies 2T10 directed against HPyV7-sTAg and 1T1 directed against HPyV6-sTAg were provided by C.Buck, NCI, Bethesda, MD, USA, and used at a 1:100 dilution in combination with the EnVision-FLEX™ visualization Kit (K8008, DAKO, Carpinteria, CA, USA) according to standard protocols [15]. 2T10-Ab is known to weakly cross-react with HPyV6, but not with MCPyV. 1T1-Ab is known to cross-react with HPyV7 and MCPyV while 2T10 Ab is known to weakly cross-react with HPyV6 [22].

In addition, the CM2B4 monoclonal antibody was used to detect LT-antigen expression of MCPyV, which is more sensitive and specific than 2T10 and 1T1 Ab as described by Moshiri et al. (clone: CM2B4, dilution 1:50; Santa Cruz Biotechnology Inc., Santa Cruz, CA, USA) [23]. The protocol used for HPyV6 and HPyV7 IHC was described previously, as was the protocol used for CM2B4 MCPyV IHC [15,24].

HPyV7-positive thymoma tissues were used as previously published [15]. Keratoacanthoma tissue positive for HPyV6 was used as a control for 1T1 IHC [18]. MCC cell line MKL-1 positive for MCPyV was used as a positive control for the CM2B4 antibody. All slides were evaluated by three pathologists (I.S., V.W., and A.z.H.) and photos were taken from the scans obtained by the VENTANA iScan-HT slide scanner. IHC was performed in selected PCR virus-positive cases and randomly for selected PCR virus-negative cases.

The immunohistochemistry stainings were scored either microscopically or using digital slides. The whole tissue sections were evaluated for the presence of positive nuclei and cytoplasm in hepatocytes, large bile duct, portal tracts, and neoplastic CCA cells. The percentage of positive cells was assessed. Inflammatory infiltrate was also evaluated if it was present. The intensity of HPyVs staining was evaluated and scored as strong (+++) if the positive nuclei were seen at 100× magnification, moderate (++) if the positive nuclei were seen at 200× magnification and weak (+) if positive nuclei were seen at 400× magnification.

In all subanatomic histological compartments (hepatocytes, large bile duct, and tumor cells), the quantitative analysis of HPyV7 IHC positive cells was evaluated using morphometry by (I.S., F.K.) on digital slides. The slides were scanned with the VENTANA iScan-HT slide scanner (Roche Diagnostics Inc., Tucson, AZ, USA) at 400× magnification. The morphometry was performed by calculating positive nuclei in the epithelial lining of the portal bile ducts, hepatocytes, and tumor cells in the hotspots in 5 photos at 400× magnification (HPF, high power field). The average of positive nuclei in the five images was calculated, and the results were present as a number of positive cells per HPF.

### 2.7. Statistical Analysis

The overall survival (OS) curve was estimated using the Kaplan–Meier method, and differences between HPyV-positive and -negative CCA cases were evaluated using a log rank test. OS was defined as the time between the date of tumor biopsy and date of death. Follow-up data for 39 (93%) patients were available, of which two patients were excluded due to the lack of survival data, only iCCA and pCCA were selected. Statistical analysis was performed with R-studio, and a *p*-value of <0.05 was considered to be statistically significant. The cohort was used to build the survival model. For feature selection, we first removed correlated features (Pearson’s r > 0.90), using the publicly available Caret package [25]. The remaining features were used to build a Cox proportional hazards model, using the publicly available rms package [26]. The hazards ratio was calculated from the regression coefficients.

## 3. Results

### 3.1. Histopathology and Specimen Quality

All isolated DNAs of the FFPE blocks revealed sufficient DNA quality with PCR products > 300 bp based on the results of the specimen control size (SCS) ladder PCR (Supplementary Figure S1a). A consensus histopathological diagnosis by three pathologists revealed that thirty (71.4%) patients had intrahepatic-CCA (iCCA), eleven (26.2%) had perihilar-CCA (pCCA), and one (2.4%) had distal-CCA (dCCA). No virus-like cytopathic effects or changes were observed in the cholangiocytes or hepatocytes during the review of HPyVs positive patient tissue specimens. Overall, 55% of patients were diagnosed at stage I, 26% at stage II, 14% at stage III, and 5% at stage IV.

### 3.2. Screening for Human Polyomaviruses Using Degenerate and HPyVs-Specific PCR

**Figure 1:**
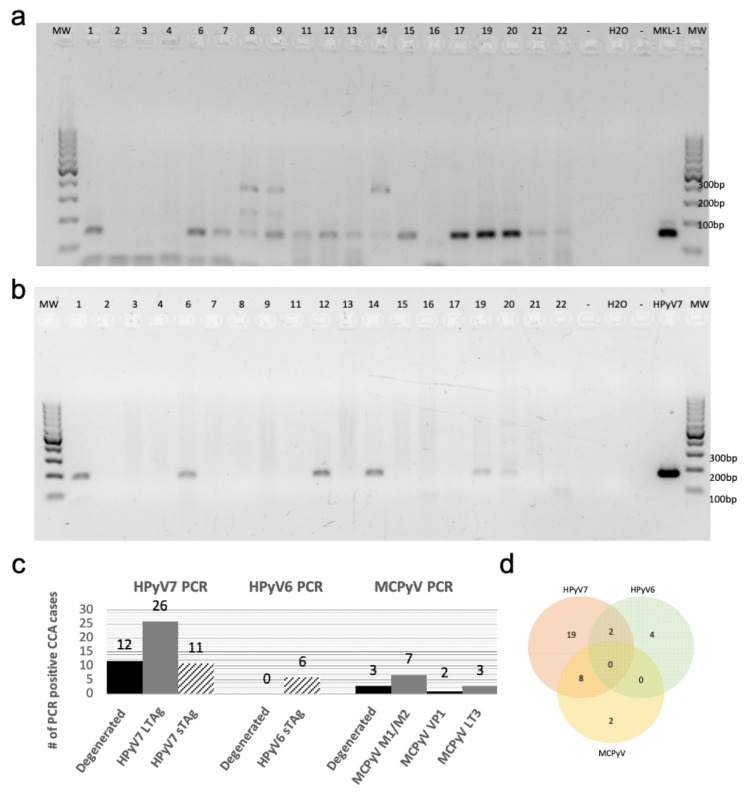
Degenerated and specific HPyV7 DNA-PCR. (**a**) The results of screening for HPyVs using FFPE tissues of Cholangiocarcinoma (CCA) by performing degenerated PCR-DNA. (**b**) Specific DNA-PCR amplifying HPyV7 sTAg (181 bp). (**c**) A chart summarizing the total HPyV results for both degenerated and specific DNA-PCR. The numbers on the gels refer to the number of CCA tissues of the respective CCA patients. (**d**) Venn diagram showing cases with two different HPyVs simultaneously. Abbreviations: Numbers on gels: represents the patient ID; H_2_O: water non-template negative control; MKL-1: MCPyV-positive Merkel cell carcinoma cell line used as a positive control; empty slot (no mastermix added to the gel); MW: molecular weight marker.

We used the MCPyV-positive MKL-1 cell line and diverse polyomaviruses (PyVs) plasmids as positive controls for PCR with the modified degenerated primers. Accordingly, the modified degenerated primers were able to amplify PyVs sequences from MCPyV, HPyV6, 7, 9, NJPyV, WUV, CaPyV, and BPyV plasmids as well as the MKL-1 cell line (Supplementary Figure S1b), as confirmed by subsequent sequencing of PCR products. Screening of the first 19 CCA cases with this PCR technique revealed that fifteen (79%) cases were positive for one of the novel HPyVs as confirmed by sequencing. HPyV7 DNA was detected in twelve CCA cases (63%), followed by MCPyV DNA in three (16%) cases. Of interest, no HPyV6 DNA was detected in the CCAs using degenerated PCR (Figure 1a).

Using HPyV7-specific PCR for all CCA cases of the cohort, 62% (26/42) of CCA tissues were tested positive for the LTAg in comparison to 26% (11/42) CCAs which were tested positive for the sTAg DNA-PCR (Figure 1b) (Supplementary Figure S2a,b). In contrast, only 14% (6/42) of cases were positive by HPyV6-specific PCR, which were negative in the degenerated primer PCR. MCPyV-specific PCR revealed seven cases (17%) positive for the tumor antigen common region (M1/M2), two (5%) positive for the viral protein gene 1 (VP1), and three (7%) for the LTAg gene (LT3) (Supplementary Figure S2c,d). All PCR products were confirmed by sequence analysis, with few nucleotide differences between the cases (Table 1).

In summary, the results of all PCR approaches revealed that 59% (25/42) of CCA tissues were positive for one of the HPyVs, while 10 (24%) CCA tissues were positive for 2 HPyVs, and 7 (17%) CCA tissues were negative for any HPyVs. Of the total 35 positive CCA tissues, 19 (45%) were positive for HPyV7, 4 (9%) for HPyV6, 2 (5%) for Merkel cell polyomavirus (MCPyV), 8 (19%) for both HPyV7/MCPyV, and 2 (5%) for both HPyV6/HPyV7 (Figure 1c,d). Combined, the results of the HPyV7-specific and modified degenerate primer PCR showed that 29 (69%) CCA tissues were positive for HPyV7 as confirmed by sequencing. Degenerated primer PCR showed 3 (16%) CCA tissues positive for MCPyV after sequencing. Both degenerated and MCPyV-specific PCR combined detected 10 (24%) CCA tissues positive for MCPyV (Table 1). In this CCA cohort, we did not observe an association of a specific HPyV with a specific CCA subtype. Only one HPyV type was detected in the samples of each CCA subtype, except for iCCA in which 10 (24%) CCAs were simultaneously harboring two different HPyVs. Eight cases were coinfected with HPyV7 and MCPyV, and two cases were coinfected with HPyV6 and HPyV7 (Figure 1d).

### 3.3. Fluorescence In Situ Hybridization (FISH)

Selected FFPE tissues chosen for assessing the FISH pretreatment with the alpha satellite (centromeric) DNA probe quality revealed intense FISH signals in all parenchymal and non-parenchymal liver cells. Thus, the protocol was optimal and was used for subsequent HPyV FISH analyses without modifications (Supplementary Figure S3a).

**Figure 2:**
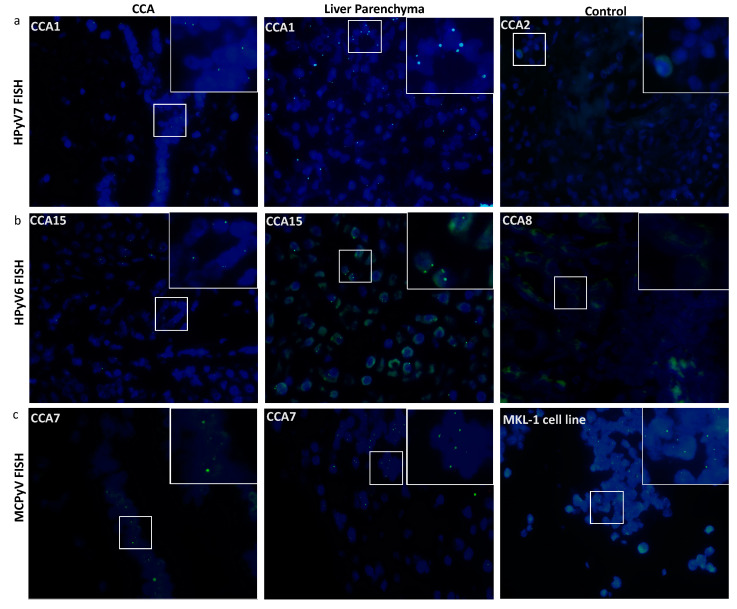
Detection of HPyV6, HPyV7, and MCPyV on the DNA in FFPE of CCA and non-neoplastic hepatocytes. Merged green (FITC) and blue (nuclei were counterstained with DAPI) show specific green signals. (**a**) Whole HPyV7 genome FISH probe performed on CCA1 tissue as a representative example of FISH for HPyV7 in CCA and non-neoplastic liver parenchyma cell while CCA2 tissue represents a negative case for HPyV7 DNA. (**b**) Whole HPyV6 genome FISH probe showed positive signals in both CCA and hepatocytes of CCA15, while the PCR-negative CCA8 case tissue revealed no signals. (**c**) Example of results of FISH specific nuclear of MCPyV in the nuclei of CCA epithelial, and the non-neoplastic hepatocytes. MKL-1 cell lines served as a positive control for the MCPyV probe. The images were taken at 630x magnification, a white square area was magnified 6x in the top right corner of each figure.

Twelve CCA tissues were subjected to HPyV7-specific FISH analysis and four of them revealed specific nuclear signals in CCA tumor cells as well as non-neoplastic bile duct epithelium and hepatocytes (Table 2). HPyV7 DNA was detected as weak to moderate nuclear punctate signals in neoplastic CCA cells, and as moderate to strong punctate nuclear signals in non-neoplastic hepatocytes (Figure 2a). To assess if the FISH signals are DNA-specific, FISH analysis using the full length HPyV7 probe was performed on three tissue slides of case CCA1 (CCA patient with ID number 1). The first slide was hybridized with the HPyV7 probe resulting in HPyV7-specific green signals in the bile duct tumor tissues as well as non-neoplastic hepatocytes. Pretreatment of the second slide with RNAse reduced the FISH signal intensity, while DNAse pretreatment diminished the specific signals almost completely as have been seen with the positive control (Supplementary Figure S3b).

All three HPyV6 DNA PCR positive cases showed specific nuclear HPyV6 DNA signals after FISH, although the dots were observed more often in the hepatocytes with normal morphology than in the CCA cells (Figure 2b). Additionally, the presence of MCPyV was confirmed by FISH in four out of five MCPyV DNA positive PCR cases (Figure 2c) (Table 2).

### 3.4. RNA In Situ Hybridization (RISH)

**Figure 3:**
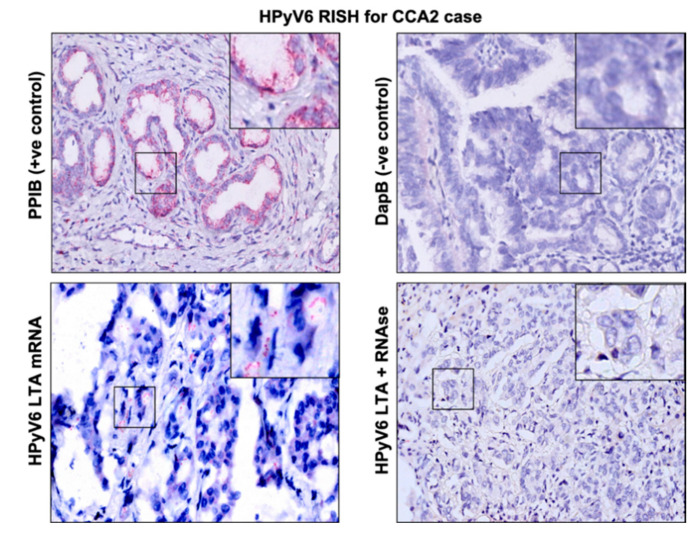
Detection of HPyV6 on the transcriptional level in FFPE CCA tissue by RNA-ISH, the CCA2 patient tissue section was hybridized with 20 sets labeled probes to detect HPyV6 LTAg mRNA using RNAscope RNA in situ hybridization assay. Additionally, FFPE hybridized with both the bacterial gene (DapB) as a negative control and the housekeeping gene (PPIB) as a positive control. Positive red signals were detected using fast red chromogen. HPyV6 LTA transcript seen as red signals in CCA and hepatocytes. No HPyV6 RNA was seen when the tissue was treated with RNAse, nuclei were counterstained with hematoxylin. The images were taken at 200x magnification, a black square area was magnified 6x in the top right corner of each figure.

The RNAscope technique was applied on 5 CCA tissues (2 HPyV6-positive CCA tissues and 3 HPyV6-negative CCA tissues, Table 2). The selection of these was based on the DNA PCR results and the availability of tissue specimens. HPyV6 mRNA was detected as specific red punctate spots in the nucleus and/or cytoplasm in two cases in both non-neoplastic hepatocytes and CCA cells, which had also tested positive by specific sTAg-PCR, FISH, and immunohistochemistry (IHC) (Figure 3). Unfortunately, RNAscope for HPyV7 mRNA detection did not work in our hands. The background ratio in the selected tissues made an interpretation of specific HPyV7 mRNA signals impossible.

### 3.5. Detection of HPyV7-sTAg, HPyV6-sTAg, and MCPyV-LTag Expression by Immunohistochemistry

**Figure 4:**
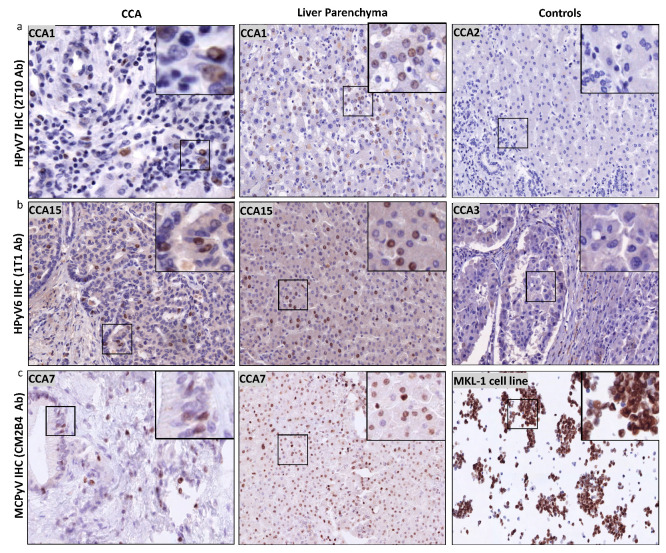
Detection of HPyV6, HPyV7, and MCPyV on the translational level in FFPE of CCA and non-neoplastic hepatocytes. (**a**) Representative examples of IHC using 2t10 antibodies showed the specific nuclear expression in the nucleus (brown) of both CCA and liver parenchyma. CCA2 tissue is an example of negative HPyV7. (**b**) In a representative IHC for CCA15 tissue, 1t1 antibodies show positivity in both CCA and hepatocytes, while no protein expression was seen in CCA3. (**c**) Example of results of IHC for MCPyV, specific nuclear expression (brown) of MCPyV (CM2B4 antibody) in the nuclei of CCA epithelial, and the non-neoplastic hepatocytes. MKL1 cell line served as a positive for MCPyV antibodies. The images were taken at 200x magnification, a black square area was magnified 6x in the top right corner of each figure.

Twelve of sixteen CCA tissues were tested positive by HPyV7-IHC. Nine CCA tissues expressed HPyV7-sTAg in tumor cells, three of which showed strong nuclear expression in at least 20% of the tumor cells (3/9; 33%) and six showed moderate to weak nuclear expression in five–ten percent of the tumor cells (Figure 4a). The expression of HPyV7-sTAg within the histomorphological non-neoplastic adjacent large and small bile duct epithelium was also assessed and revealed nuclear positivity in eleven cases (11/16; 69%) (Table 2). No strong expression was observed, and seven cases showed weak expression in less than 5% of bile ducts. Of interest, adjacent histomorphological non-neoplastic liver parenchyma also revealed specific nuclear positivity for HPyV7 in 11 cases (11/6; 65%) (Figure 4a). Strong hepatocellular expression was seen in three cases, moderate intensity in one case, and weak staining in seven cases (Table 2). The percentage of positive nuclei varied from less than 5% up to approximately 80%. No cytoplasmic expression was observed in neoplastic or non-neoplastic bile duct epithelium and hepatocytes, and no differences in zonal distribution were observed. In cases with a prominent inflammatory infiltrate, scattered lymphocytes also showed nuclear positivity for HPyV7. Furthermore, endothelium of the blood vessels was completely negative for HPyV7-sTAg expression.

Moreover, the number of HPyV7-positive nuclei in hepatocytes, bile duct epithelium, and tumor cells was counted in the hotspots in 5 high power fields (HPF; 400X) using morphometric analysis (Table S1). Thus, these findings confirm at least a low HPyV7 protein expression in at least a few positive cells per HPF in all positive IHC cases of CCA regardless of the CCA subtype and/or clinical stage. Moreover, periportal hepatocytes showed at least a focal positivity in all of these cases. In cases with reactive ductular reaction within the limiting plate of the portal tract, the newly formed small ducts also showed at least focal immunoreactivity.

Twelve of the selected fourteen CCA tissues were tested positive for HPyV6-sTAg IHC expression. However, more CCA tissues revealed HPyV6-IHC positivity than PCR due to the fact that 1t1 antibodies are well known to have cross-reactivity with HPyV7 and MCPyV. The intensity of the HPyV6-IHC staining was weak to moderate with a maximal 10% of positive cells (Figure 4b). No sTAg expression within the cytoplasm was observed, and no expression was found in endothelial cells of blood/lymphatic vessels. Of interest, twelve CCA tissues revealed specific nuclear HPyV6-sTAg expression in non-neoplastic hepatocytes (12/14; 86%). Six cases revealed strong nuclear reactivity with percentages of positive nuclei between 20% and 80% (Figure 4b). No differences in zonal distribution were observed. Normal epithelial lining of bile duct epithelium was only weakly positive in 5 to 40% of the nuclei of cholangiocytes.

Six CCA tissues were tested for MCPyV-LTag expression by IHC, five of which showed nuclear staining (5/6; 83.3%) (Figure 4c). Three CCA tissues revealed both weak nuclear positivity in maximal 30% of tumor cells. All of the hepatocytes showed a weak to moderate staining pattern (Figure 4c). One CCA tissue revealed cytoplasmic staining, which was interpreted as unspecific and thus negative. No differences in zonal distribution were seen. Normal epithelial lining of bile duct epithelium was only positive in three cases up to 30% of nuclei of bile duct epithelium (ranging between weak and strong intensity). No cytoplasmic staining was observed (Table 2).

### 3.6. Overall Survival Analysis

**Figure 5:**
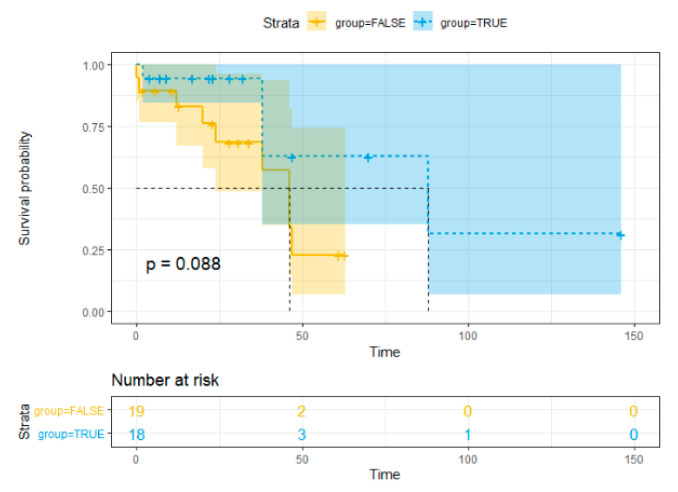
Kaplan–Meier method survival curve for overall survival.

Statistics data analysis revealed no significant association between overall survival (OS) and HPyV7 (*p* = 0.726), HPyV6 (*p* = 0.366), and MCPyV (*p* = 0.953) in iCCA and pCCA. The hazards ratio and the *p*-value are reported in Cox regression data (Table 3). The Kaplan–Meier curve showed no significant risk stratification for CCA patients (Figure 5).

## 4. Discussion

The etiology of CCA is largely unknown. Here, we investigated the presence of HPyV7, 6, and MCPyV in CCA tissue specimens, including adjacent histomorphological non-neoplastic bile duct epithelium and non-neoplastic hepatocytes, using diverse molecular detection techniques. We used a combined approach of a degenerated primer set for HPyVs and diverse HPyVs-specific PCRs, all PCRs were followed by sequence analysis. Degenerated PCR revealed to be a reliable detection of HPyVs in FFPE tissues demonstrating that the use of degenerated HPyV primers is applicable for screening purposes of FFPE tissues. (Figure 1a). To the best of our knowledge, this is the first study to perform FISH and IHC to confirm the presence of HPyV6, HPyV7, and MCPyV in the bile duct epithelium and hepatocytes, and also to show HPyV6 transcript by RNA-ISH in human tissues.

Compared to the prevalence of HPyV6 DNA in CCA tissues of our cohort 6 (14%), HPyV7 DNA was detected much more frequently in 29 (69%) of the CCA tissues. Of interest, some of the cases that tested HPyV7 DNA positive by degenerate LTAg PCR were negative by specific HPyV7 DNA-PCR targeting sTAg. However, assessing the whole cohort with a specific LTAg HPyV7 DNA-PCR within the conserved region which is targeted by the degenerated primers revealed a good agreement (Figure S2). This apparent incongruency of HPyVs DNA (LTAg vs sTAg) detection by PCR in FFPE tissue using different primer pairs is a well-known phenomenon. It is most likely due to the abundance of the diverse targeted sequences of the viral genome and is in line with prior observations of us and others in the detection of HPyVs such as MCPyV, HPyV6, and HPyV7 [4,11,27,28]. We have proven this discrepant phenomenon by using two specific HPyV7-DNA PCR targeting different regions (sTAg and LTAg). Our results showed that the rate of detection of specific HPyV7 LTAg is higher than the detection of sTAg, matching the results of the degenerated PCR.

An explanation for the discrepancy between high HPyV7 prevalence found by us and high HPyV6 prevalence found by Chan et al. might be the primary source of the DNA, i.e., primary CCA tissues versus bile fluids of CCA patients, respectively, or due to variation in the ethnical and geographical distribution of both cohorts. Recent studies have assessed HPyVs serum antibody levels across a range of age groups including one from the Netherlands. HPyV7 and HPyV6 were found to be highly seroprevalent in all age groups [29,30,31,32,33]. HPyV6 seroprevalence was slightly higher than HPyV7 and MCPyV. However, the mean age in our patient cohort was 65.2 years compared to 77 years in the study of Chan et al., largely ruling out age related effects to explain these diverse findings.

In addition to HPyV7 specific DNA PCR, we were able to visualize the presence of HPyV7 by FISH and IHC. The FISH results were in good agreement with the PCR results and allowed the identification of the virus at the single cell level within the histomorphological context. FISH revealed the presence of HPyV7 within non-neoplastic hepatocytes and bile duct epithelium as well as CCA tumor tissue (Figure 2a). However, not all tumor cells revealed the presence of HPyV7 DNA by FISH. On the protein level using the 2t10 IHC antibody, nine CCA cases revealed an accumulation of the HPyV7 sTAg in tumor cells. Compared to non-neoplastic bile duct epithelium as well as adjacent non-neoplastic hepatocytes, eleven cases showed expression of HPyV7 sTAg. Finding less expression in CCA tumor cases might be explained by the fact that CCA can be derived from different cellular origins, including mature hepatocytes [34,35]. Due to the different origins of cholangiocytes, we have seen some CCAs which were positive and some which were negative. Therefore, one can speculate that HPyV7 might infect hepatocytes at an early stage, considering that hepatocytes have the potential to differentiate between bile ducts or CCA. CCA cells with negative HPyV7 expression might differentiate from the non-hepatocyte origin.

Unfortunately, we were unable to detect specific mRNA signals for HPyV7 RISH due to technical reasons, which could be explained by two possibilities: the background ratio in the selected tissues made an interpretation of specific HPyV7 mRNA signals impossible; or the selected target sequences of the HPyV7 probe need to be adapted.

The above findings indicate that HPyV7 is most probably not directly related to the etiopathogenesis of CCA. However, the presence of HPyV7 in only a part of the tumor cells may also have been caused by technical restrictions of the FISH and IHC assays. The detection sensitivity of these assays is dependent on optimal pre-analytic tissue processing and preservation of nucleic acid quality and protein epitopes, which sometimes can be limited in FFPE tissues.

In the underlying study, a relatively low 14% prevalence of HPyV6 was detected in CCA tissues by HPyV6 specific PCR, which is lower than the 27% reported by Chan et al. in the bile fluid of CCA patients. By using the RNAscope technique, we were able to demonstrate HPyV6 transcripts on the single cell level in both non-neoplastic hepatocytes and CCA cells. This is in line with the DNA FISH and sTAg IHC results. The IHC results, however, need to be interpreted with care, since we also detected the HPyV6 sTAg expression in HPyV6 PCR-negative but HPyV7- and MCPyV- PCR positive cases, which is most likely due to the known cross-reactivity of this antibody with HPyV7 and MCPyV [22].

MCPyV was found in 24% of CCAs by both degenerated and specific DNA PCR, which is consistent with previous reports [36,37]. Loyo and colleagues reported the presence of MCPyV DNA by qPCR in five (33%) out of fifteen normal liver tissues, ten (63%) out of sixteen liver cancers, and two (100%) out of two biliary cirrhosis cases [36]. Additionally, the prevalence of MCPyV has been reported in three (24%) of 124 nonmalignant liver tissues by generic polyomavirus PCR [37]. MCPyV FISH analysis was able to detect the virus DNA in CCA tumors as well as non-neoplastic hepatocytes and bile duct epithelium (Figure 2c), showing relatively weak signals similar to the general weak to faint amplicons detected by PCR. On the protein level, weak immunostaining of MCPyV LTAg viral proteins within both the CCA neoplastic cell and the normal looking bile duct epithelium was also observed. Thus, all three detection methods provided evidence for the presence of MCPyV in a subset of CCAs and normal liver and bile duct tissue. It is well known that malignant tumors associated with DNA viruses have a better survival as reported previously [38,39]. Our study revealed that iCCA and pCCA carrying HPyV7, HPyV6, or MCPyV DNA did not predict an association with overall survival and further larger studies are needed to confirm our findings.

The contribution of HPyV 7 to CCA carcinogenesis cannot be definitely elucidated by the present data. However, it remains speculation in as much as HPyV7 possibly contributes to CCA carcinogenesis. This might be based on a direct HPyV7-interaction and subsequent deactivation of p53, as recently reported by Rozenblatt-Rosen et al. [40], or alternatively, by clonal integration of the HPyV7 genome with or without tumor-specific viral mutations as has been shown for MCPyV-positive MCCs [11,41]. The latter seems to be rather unlikely for HPyV7-positive CCAs as one would then expect a high expression of viral proteins in every tumor cell. The most likely contribution of HPyV7 and maybe other HPyVs is an indirect mechanism via the induction of (chronic) inflammation as has been convincingly shown for Hepatitis B and C viruses [1].

There are some limitations to our study. These limitations are mainly due to the rarity of CCA which also explains the sample size of our patient cohort (*n* = 42). Of course, it would be of clinical interest to further elucidate possible other correlations between these HPyVs and clinical endpoints of CCA.

## 5. Conclusions

Our results strongly indicate that HPyV7, HPyV6, and MCPyV infect non-neoplastic hepatocytes, bile duct epithelium, and CCA lesions. Although all three HPyVs are hepatotropic, HPyV7 was more frequently detected in both neoplastic and non-neoplastic cells in our CCA subset compared to HPyV6 and MCPyV. To the best of our knowledge, this is the first study to report the presence of HPyV7, HpyV6, and MCPyV on the single cell level in liver tissues of non-neoplastic bile duct epithelium and hepatocytes and CCA. We used diverse molecular techniques to assess the presence of these viruses. Although our results might point to indirect role of these HPyVs to CCA carcinogenesis, possibly by inflammation, the role of these HPyVs in the etiopathogenesis of CCA needs to be further elucidated. Furthermore, the frequent finding of HPyVs, especially HPyV7 in liver tissues might also point to a possible role of HPyV7 in other hepatocellular diseases.

## Figures and Tables

**Table 1:** Clinical data and DNA PCR results.

Patient ID.	Gender	Age	Diagnosis	Clinical Stage	Degenerated PCR	HPyV7		HPyV6	MCPyV			Result
						LTAg	sTAg	sTAg	M1/M2	VP1	LT3	
**CCA1**	F	67	iCCA	II	HPyV7	+	+	−	+	+	+	HPyV7/MCPyV
**CCA2**	M	59	iCCA	II	−	−	−	+	−	−	−	HPyV6
**CCA3**	F	68	iCCA	I	−	−	−	−	−	−	−	−
**CCA4**	F	29	iCCA	III	−	−	−	+	−	−	−	HPyV6
**CCA6**	M	74	iCCA	I	MCPyV	+	+	−	−	−	−	HPyV7/MCPyV
**CCA7**	M	64	pCCA	I	MCPyV	−	−	−	−	−	+	MCPyV
**CCA8**	F	64	pCCA	III	HPyV7	+	−	−	−	−	−	HPyV7
**CCA9**	M	45	pCCA	I	MCPyV	−	−	−	+	−	−	MCPyV
**CCA11**	F	50	dCCA	II	HPyV7	+	−	−	−	−	−	HPyV7
**CCA12**	M	70	iCCA	II	HPyV7	+	+	−	−	−	−	HPyV7
**CCA13**	M	69	iCCA	I	HPyV7	+	−	−	−	−	−	HPyV7
**CCA14**	M	70	iCCA	I	HPyV7	+	+	−	−	−	−	HPyV7
**CCA15**	F	71	iCCA	I	HPyV7	+	−	+	−	−	−	HPyV6/HPyV7
**CCA16**	F	64	iCCA	II	−	−	−	−	−	−	−	−
**CCA17**	M	59	pCCA	III	HPyV7	+	−	−	−	−	−	HPyV7
**CCA19**	M	69	iCCA	I	HPyV7	+	+	−	+	−	+	HPyV7/MCPyV
**CCA20**	M	71	iCCA	II	HPyV7	+	+	−	−	−	−	HPyV7
**CCA21**	F	63	iCCA	III	HPyV7	+	−	−	−	−	−	HPyV7
**CCA22**	M	61	iCCA	I	HPyV7	+	−	−	−	−	−	HPyV7
**CCA23**	M	63	iCCA	I	NA	+	−	−	−	−	−	HPyV7
**CCA24**	M	73	iCCA	I	NA	+	−	−	+	−	−	MCPyV/HPyV7
**CCA25**	F	74	iCCA	II	NA	−	−	+	−	−	−	HPyV6
**CCA26**	F	60	iCCA	II	NA	+	+	−	−	−	−	HPyV7
**CCA27**	M	75	iCCA	I	NA	+	−	−	+	−	−	MCPyV/HPyV7
**CCA28**	M	50	pCCA	I	NA	+	−	−	−	−	−	HPyV7
**CCA29**	F	45	iCCA	I	NA	−	−	−	−	−	−	−
**CCA30**	F	66	pCCA	I	NA	−	+	−	−	−	−	HPyV7
**CCA31**	F	74	iCCA	III	NA	+	+	−	−	−	−	HPyV7
**CCA32**	F	77	iCCA	I	NA	+	−	−	+	−	−	MCPyV/HPyV7
**CCA33**	M	77	iCCA	IV	NA	+	−	−	−	+	−	MCPyV/HPyV7
**CCA34**	M	77	pCCA	I	NA	−	−	+	−	−	−	HPyV6
**CCA35**	M	59	pCCA	IV	NA	−	+	−	−	−	−	HPyV7
**CCA36**	M	72	pCCA	II	NA	+	−	−	−	−	−	HPyV7
**CCA37**	M	60	iCCA	I	NA	−	+	+	−	−	−	HPyV7/HPyV6
**CCA38**	F	85	iCCA	I	NA	−	−	−	−	−	−	−
**CCA39**	M	52	iCCA	II	NA	+	−	−	−	−	−	HPyV7
**CCA40**	M	63	iCCA	II	NA	−	−	−	−	−	−	−
**CCA41**	M	78	pCCA	I	NA	−	−	−	−	−	−	−
**CCA42**	F	72	iCCA	I	NA	+	−	−	−	−	−	HPyV7
**CCA43**	F	74	iCCA	I	NA	+	−	−	+	−	−	MCPyV/HPyV7
**CCA44**	M	60	iCCA	III	NA	−	−	−	−	−	−	−
**CCA45**	M	66	pCCA	I	NA	+	−	−	−	−	−	HPyV7
Results	F:17 M:25		iCCA:30 pCCA:11 dCCA:1		HPyV7:12 MCPyV:3	26/42	11/42	6/42	7/42	2/42	3/42	HPyV7:29 HPyV6: 6 MCPyV:10

Abbreviations: Patient ID, lab identification number; CCA, Cholangiocarcinoma; iCCA, intrahepatic; pCCA, perihilar; dCCA, distal; PCR, Polymerase chain reaction; +, positive; −, negative; NA, not applicable; HPyV6, human polyomavirus 6; HPyV7, human polyomavirus 7; MCPyV, Merkel cell polyomavirus; sTAg, small tumor antigen; LTAg, large tumor antigen; M1/M2, common region between sTAg and LTAg; VP, viral protein.

**Table 2:** Fluorescence in situ hybridization, immunohistochemistry, and RNA in situ hybridization results.

Patient ID	HPyV7				HPyV6			MCPyV		Result
	FISH	IHC			FISH	RNA-Ish	IHC	FISH	IHC	
		Hepatocyte	Bile Duct Epithelium	CCA						
**CCA1**	++	+++	++	++	−	NA	+++	+	+	HPyV7 MCPyV
**CCA2**	−	−	−	−	+	+	++	−	NA	HPyV6
**CCA4**	−	NA	NA	NA	+	NA	+++	NA	NA	HPyV6
**CCA6**	−	+	++	−	−	NA	++	+	++	HPyV7 MCPyV
**CCA7**	NA	−	−	−	−	−	+++	++	+++	MCPyV
**CCA8**	−	+	+	+++	−	−	++	NA	NA	HPyV7
**CCA9**	−	−	−	−	−	NA	+++	−	++	MCPyV
**CCA11**	−	++	++	+++	−	NA	NA	NA	−	HPyV7
**CCA12**	−	+	++	+	−	NA	NA	NA	NA	HPyV7
**CCA13**	NA	−	−	+	NA	NA	NA	NA	NA	HPyV7
**CCA14**	+	+++	+	−	NA	−	+++	NA	NA	HPyV7
**CCA15**	+	+++	+	+++	++	+	+++	NA	NA	HPyV6 HPyV7
**CCA16**	NA	−	−	−	NA	NA	−	−	NA	−
**CCA17**	NA	+	+	−	NA	NA	+	NA	NA	HPyV7
**CCA19**	NA	+	+	+	NA	NA	−	+	+	HPyV7 MCPyV
**CCA20**	NA	+	+	+	NA	NA	+	NA	NA	HPyV7
**CCA21**	+	+	+	+	−	NA	+	NA	NA	HPyV7
**Total**	4/11	11/16	11/16	9/16	3/11	2/5	12/14	4/7	5/6	

Abbreviations: Patient ID, lab identification number; CCA, Cholangiocarcinoma; +, weak positive; ++, moderate positive; +++, strong positive; −, negative; NA, not applicable; HPyV6, human polyomavirus 6; HPyV7, human polyomavirus 7; MCPyV, Merkel cell polyomavirus; sTAg, small tumor antigen; LTAg, large tumor antigen; IHC, immunohistochemistry; FISH, fluorescence in situ hybridization; RNA-ish, RNA in situ hybridization.

**Table 3:** Cox regression model for multivariable analysis of iCCA and pCCA cohort.

Variable	Hazards Ratio	*p*-Value
Sex	0.617888	0.521
Age	1.004683	0.893
Subtype	2.639623	0.174
Stage	0.761135	0.551
HPyV7	0.741641	0.726
HPyV6	2.293245	0.366
MCPyV	1.057001	0.953

## Supplementary Materials

The following are available online at https://www.mdpi.com/2076-2607/8/8/1125/s1. Figure S1: (a) Agarose gel showing the specimen control size (SCS) ladder protocol to validate the DNA quality to be eligible for PCR testing. (b) Representative figure for validating the modified degenerated primer set by testing diverse PyVs positive control plasmids. Figure S2: (a,b) representative gel figures of DNA-PCR for HPyV7 targeting LTAg (98 bp) for the whole cohort. (c,d) A representative gel figure of DNA-PCR for MCPyV targeting the common region of LTAg and sTAg (M1/M2—178 bp). Figure S3: validating the pretreatment of FFPE and labelled probes for FISH. Figure S4: (a,b) genome organization of HPyV6 627a (HM011563.1) showing the degenerated primers location in the LTAg (designed by genome compiler software). Table S1: morphometric analysis for HPyV7 IHC immunohistochemistry. Table S2: PCR-primers used in this study.
