# Supplementary material for: High Prevalence of Human Polyomavirus 7 in Cholangiocarcinomas and Adjacent Peritumoral Hepatocytes: Preliminary Findings
Source: Microorganisms. 2020 Jul 27;8(8):1125. doi: 10.3390/microorganisms8081125 (PMC7464213; doi:10.3390/microorganisms8081125)
Supplement: Supplementary file 1 [file microorganisms-08-01125-s001.pdf]

Supplementary:

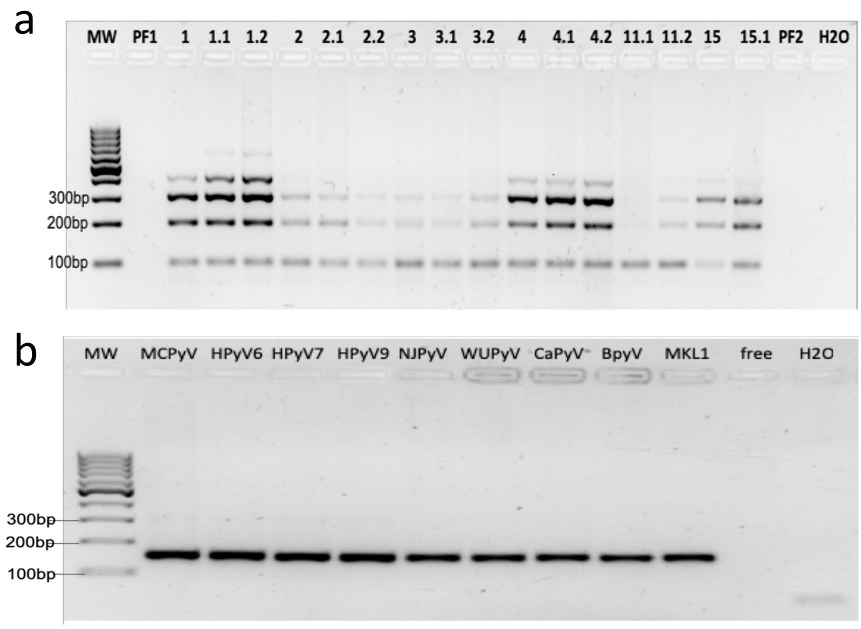

Figure S1. **(a)** Agarose gel showing the specimen control size (SCS) ladder protocol to validate the DNA quality to be eligible for PCR testing. **(b)** Representative images of DNA agarose gels for validating the modified degenerated primer set by testing diverse PyVs positive control plasmids and MKL1 cell line (142 to 158bp PCR products). **Abbreviations:** H_2O; water non-template negative control, PF; free paraffin without tissue, MW; molecular weight marker, MKL1; Merkel cell carcinoma cell line positive for MCPyV, Free; no mastermix added to the gel.

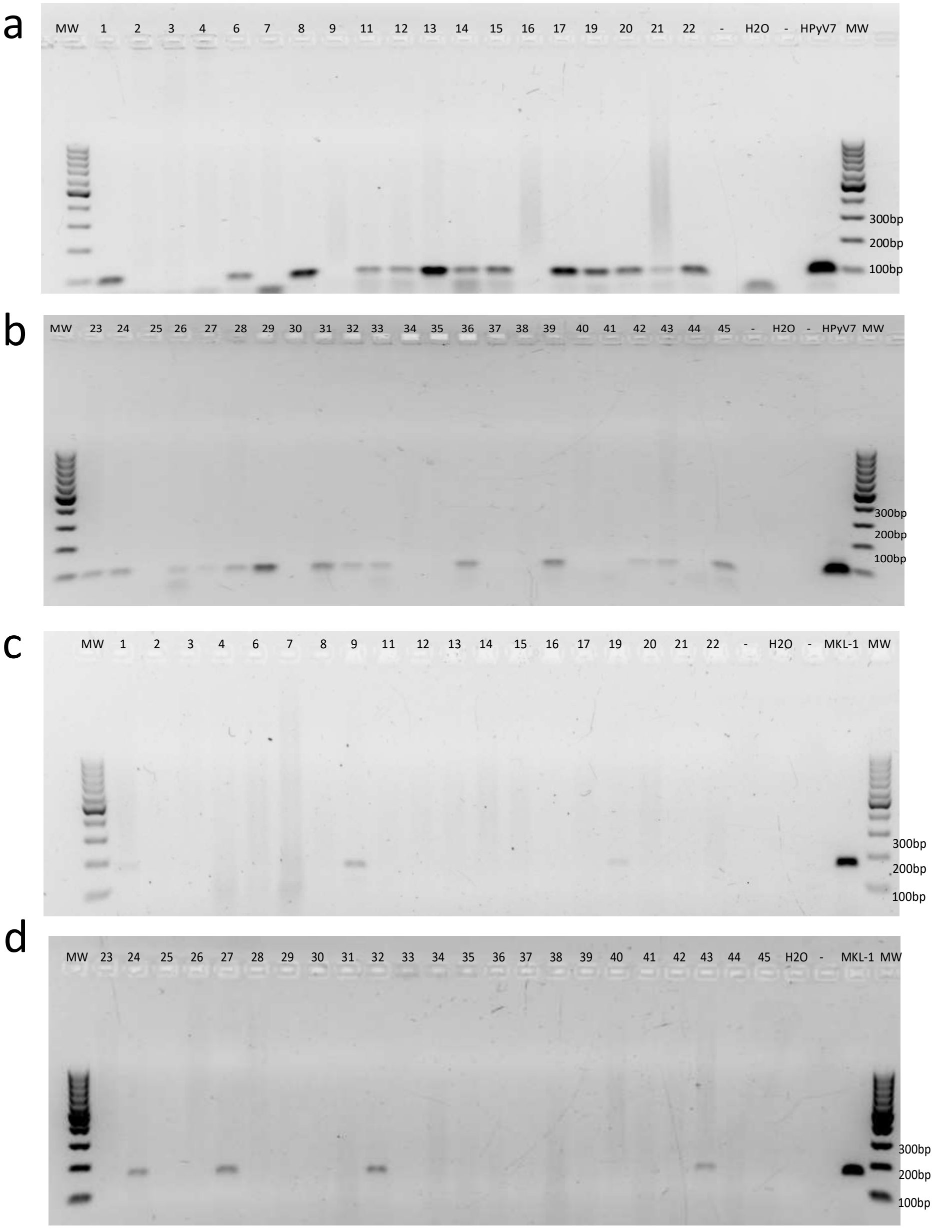

Figure S2. (a & b) A representative gel figures of DNA-PCR for HPyV7 targeting LTAg (98bp). **(c & d)** A representative gel figures of DNA-PCR for MCPyV targeting the common region of LTAg and sTAg (M1/M2 - 178bp). **Abbreviations:** H_2O; water non-template negative control, MW; molecular weight marker, HPyV7; Human polyoma virus, MKL1; Merkel cell carcinoma cell line positive for MCPyV as a positive control, - ; no mastermix added to the gel slot.

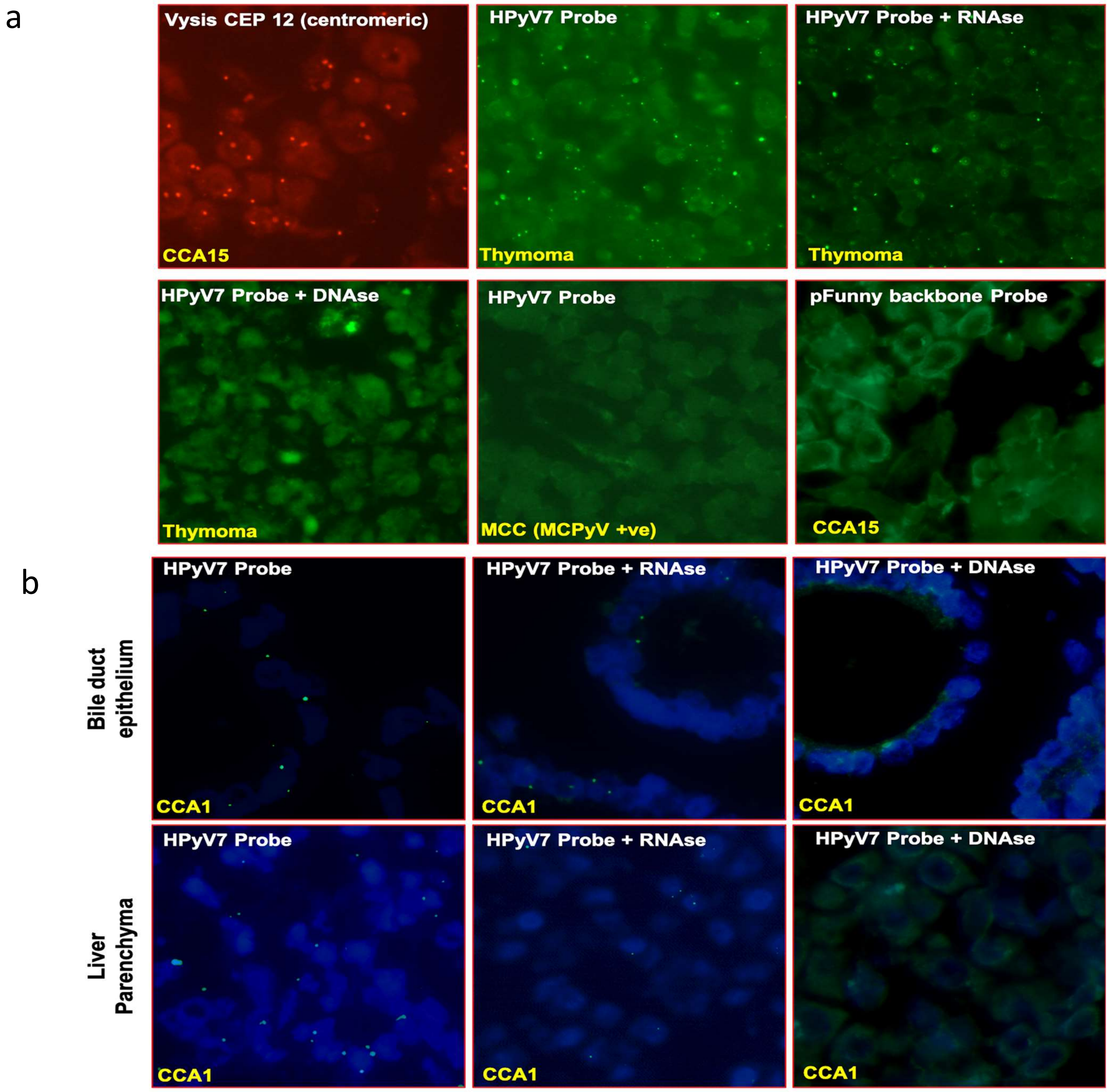

Figure S3. Validating the pretreatment of FFPE and labelled probes for FISH. (a) Validating the DNA quality and integrity for subsequent HPyV FISH analyses. Using the CEP probe directed against alpha satellite DNA located at the centromere of chromosome 12 (orange). Two copies of each signal were observed in the nuclei all parenchymal and non-parenchymal liver cells. Validating the whole HPyV7 genome probe labeling, specific green punctate dot FISH signals in thymic cells which used as control for HPyV7. HPyV7 Probe with RNase for thymoma tissues section demonstrate a slightly diminished of the fluorescence signal dots. Treatment the thymoma tissue with DNAse I in combination with the HPyV7 probe erased the signals completely. Merkel cell carcinoma tissue with positive MCPyV tested with HPyV7 probe to exclude the cross reactivity of the labeled probe with MCPyV. Testing the labeled pFunny backbone probe which used as backbone for HPyV7 plasmid and revealed no signals in the CCA and non-neoplastic hepatocyte cells which were positive for HPyV7 probe FISH. (b) Representative images for validating FISH for the HPyV7 probe using CCA tissue positive for HPyV7 by PCR, specific green punctate dot FISH signals in CCA and hepatic cells representing HPyV7 DNA. The signals were slightly decreased after RNase A treatment, however using DNase I erased the signals completely.

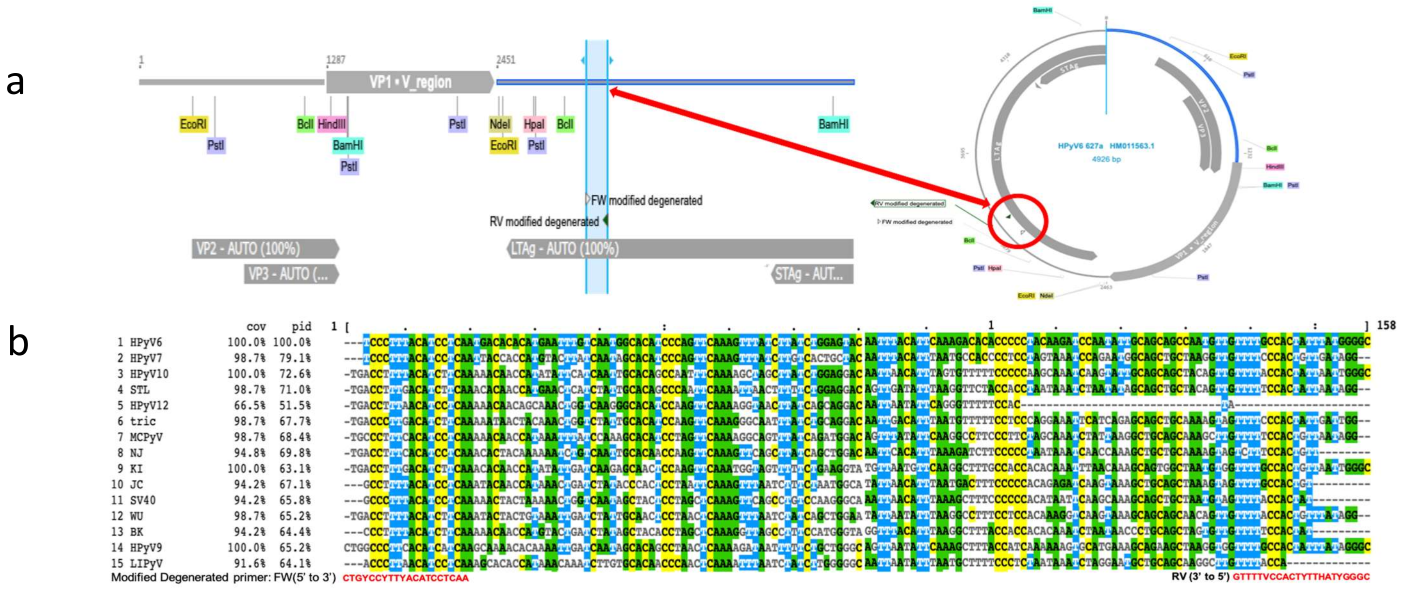

Figure S4. (a) Genome organization of HPyV6 627a (HM011563.1) showing the degenerated primers location in the LTAg (Designed by genome compiler software). **(b)** Sequence alignment of LTAg conservative region of all 14 HPyVs as well as SV40 which can be detected with the degenerated primers (designed by the Clustal Omega algorithm by The European Bioinformatics Institute).
Abbreviations: H_2O; water non-template negative control, MW; molecular weight marker.

Supplementary Table 1: Morphometric analysis for HPyV7 IHC immunohistochemistry

Patient ID	HPyV7 IHC		
	Average of positive cells by HPF		
	**Hepatocyte**	**Bile duct epith.**	**CCA**
CCA1	**23**	**18**	**11**
CCA6	**3**	**11**	**-**
CCA8	**6**	**10**	**26**
CCA11	**13**	**13**	**21**
CCA12	**4**	**12**	**2**
CCA13	**-**	**-**	**8**
CCA14	**52**	**8**	**-**
CCA15	**40**	**2**	**40**
CCA17	**1**	**1**	**-**
CCA19	**2**	**2**	**2**
CCA20	**2**	**4**	**3**
CCA21	**7**	**3**	**3**

Abbreviations: Patient ID, lab identification number; CCA, Cholangiocarcinoma; HPyV7, human polyomavirus 7; IHC, immunohistochemistry; HPF, high power field

Supplementary Table 2: PCR-primers used in this study

Primer	Gene	Forward primer (5' to 3')	Reverse primer (5' to 3')	Products size	Reference
Modified Degenerated	LTAg	CTGYCCYTTYACATCCTCAA	GCCCYATHAAYAGTGGVAAAAC	142 to 158 bp	Our group
HPyV7	sTAg	AGATTTAGCTGTCCCCAAAG (3946 – 3965)	AAGAAGGCCAAAGAGTATGC (4108 – 4127)	181 bp	(1)
HPyV7	LTAg	GTTCAAAGTTTATCTTGTCACTGC (3119 – 3142)	CAACAGTGGGAAAACAACCTTAGCA (3192 – 3216)	98 bp	Our group
HPyV6	sTAg	ATCAGCTTCCACAGGTAGGC (3438 – 3457)	TTGCCTTCTCAAAAAGGAGC (3542 – 3561)	123 bp	(1)
MCPyV	LT3	TTGTCTCGCCAGCATTGTAG (571 – 590)	ATATAGGGGCCTCGTCAACC (860 – 879)	308 bp	(2)
MCPyV	VP1	TGGATCTAGGCCCTGATTTTT (3786 – 3806)	TTTGCCAGCTTACAGTGTGG (4118 – 4137)	351 bp	(2)
MCPyV	M1/M2	GGCATGCCTGTGAATTAGGA (1711 – 1730)	TTGCAGTAATTTGTAAGGGGGCT (1867 – 1889)	178 bp	(3)

Abbreviations: HPyV6, human polyomavirus 6; HPyV7, human polyomavirus 7; MCPyV, Merkel cell polyomavirus; sTAg, small tumor antigen; LTAg, large tumor antigen; LT, large antigen; VP, viral protein; M1/M2, the common region between sTAg and LTAg; bp, base pair.

References:

1.Schowalter RM, Pastrana DV, Pumphrey KA, Moyer AL, Buck CB. Merkel cell polyomavirus and two previously unknown polyomaviruses are chronically shed from human skin. Cell host & microbe. 2010;**7(6):**509–15.

2.Feng H, Shuda M, Chang Y, Moore PS. Clonal integration of a polyomavirus in human Merkel cell carcinoma. Science (New York, NY). 2008;**319(5866)**:1096–100.

3.Kassem A, Schopflin A, Diaz C, Weyers W, Stickeler E, Werner M, et al. Frequent detection of Merkel cell polyomavirus in human Merkel cell carcinomas and identification of a unique deletion in the VP1 gene. Cancer research. 2008;**68(13)**:5009–13.
